# Top‐Down Synthesis of Wurtzite Gallium Nitride Colloidal Quantum Dots via Defect‐Driven Fragmentation

**DOI:** 10.1002/smtd.70956

**Published:** 2026-08-19

**Authors:** Yangpil Jang, Kwangwook Kim, Hayeon Baek, Dongjun Kim, Jungwon Park, Jeong Woo Han, Jongnam Park

**Affiliations:** ^1^ Department of Biomedical Engineering Ulsan National Institute of Science and Technology (UNIST) Ulsan Republic of Korea; ^2^ Department of Materials Science and Engineering Research Institute of Advanced Materials Seoul National University Seoul Republic of Korea; ^3^ School of Chemical and Biological Engineering Seoul National University Seoul Republic of Korea; ^4^ Research and Development Center UniQDot Ulsan Republic of Korea

**Keywords:** defect‐driven fragmentation, electron microscopy, quantum dots, top‐down synthesis, wurtzite gallium nitride

## Abstract

The synthesis of colloidal gallium nitride (GaN) quantum dots (QDs) is limited by the instability of organic ligands at the high GaN crystallization temperatures. Here, we report a top‐down, defect‐driven fragmentation strategy that yields high‐purity colloidal GaN QDs at 50°C and ambient pressure. This process is facilitated by nitrogen‐containing organic solvents, which we propose lower the energy barrier for native point defect segregation to the crystal surface, thereby promoting controlled structural subdivision. Defect extraction is supported by the progressive suppression of deep‐level defect luminescence and the emergence of sharp, quantum‐confined emission with a full width at half maximum of 38.8 nm. Direct observation of crystal fragmentation via identical‐location transmission electron microscopy corroborates the mechanism. Post‐synthesis analysis reveals extensive crystallite subdivision and the formation of surface Ga^2+^ states. This strategy offers a scalable route to high‐quality GaN QDs and provides a foundation for exploring the defect‐mediated synthesis of nanostructures from other chemically robust semiconductors.

## Graphical abstract

Conventional synthesis of wurtzite gallium nitride (GaN) quantum dots (QDs) demands extreme heat. We overcome this via defect‐driven fragmentation at 50°C. Nitrogen‐rich solvents provide a thermodynamic driving force that promotes the outward movement of internal defects. These migration paths evolve into structural channels, causing the bulk crystal to subdivide into high‐purity QDs.

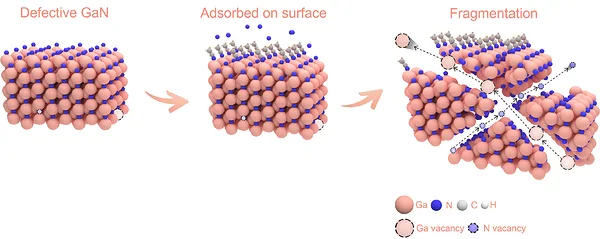

## 1 Introduction

Colloidal quantum dots (QDs) are key materials for next‐generation optoelectronics owing to their tunable electronic structures and compatibility with solution‐based processing [1, 2, 3, 4, 5, 6]. However, the synthesis of colloidal gallium nitride (GaN) QDs is significantly challenged by the incompatibility between the thermal conditions required for GaN crystallization and stable colloidal synthesis. The bond energy of GaN necessitates extreme temperatures (>500°C) and pressures for crystallization, which lead to the decomposition of organic ligands essential for stabilizing colloidal nanoparticles [7, 8, 9, 10, 11, 12, 13, 14, 15]. This thermal mismatch has largely precluded the application of conventional bottom‐up colloidal techniques to GaN. A molten salt method under ammonia at 290°C was recently introduced to synthesize colloidal GaN QDs [16]. The broad emission spectrum of the resulting QDs indicated a high density of defects, highlighting the difficulty in achieving high‐quality materials despite using state‐of‐the‐art bottom‐up approaches.

Although harsh synthesis temperatures can be avoided using top‐down fragmentation, existing methods often present considerable challenges. Mechanical approaches, such as ball milling, are limited by the extreme hardness of GaN, which complicates controlled comminution. Wet‐etching techniques used in GaN nanostructure fabrication are optimized for substrate patterning, and the fragmented byproducts have not been developed into monodisperse, optically active colloidal QDs [17, 18, 19, 20, 21]. Therefore, a chemical approach that offers better control is necessary. Inspired by defect‐driven fragmentation processes reported for other crystalline materials, we hypothesized that leveraging native point defects within GaN could provide a pathway for controlled subdivision [22, 23].

Here, we report top‐down, defect‐driven fragmentation of bulk wurtzite GaN that yields high‐quality colloidal QDs at 50°C and atmospheric pressure. We induce controlled, defect‐mediated crystal subdivision by employing nitrogen‐containing organic solvents. The resulting QDs exhibit strong, quantum‐confined excitonic emission with a photoluminescence (PL) full width at half maximum (FWHM) of only 38.8 nm and suppressed defect‐related luminescence. Structural, spectroscopic, and computational analyses support a proposed mechanism initiated by solvent‐induced defect segregation to the crystal surface. This study establishes a scalable and thermodynamically guided route to GaN QDs and introduces an adaptable strategy for the defect‐mediated nanostructuring of chemically robust group III–nitride semiconductors.

## 2 Results and Discussion

### 2.1 Synthesis and Characterization of GaN QDs

**FIGURE 1:**
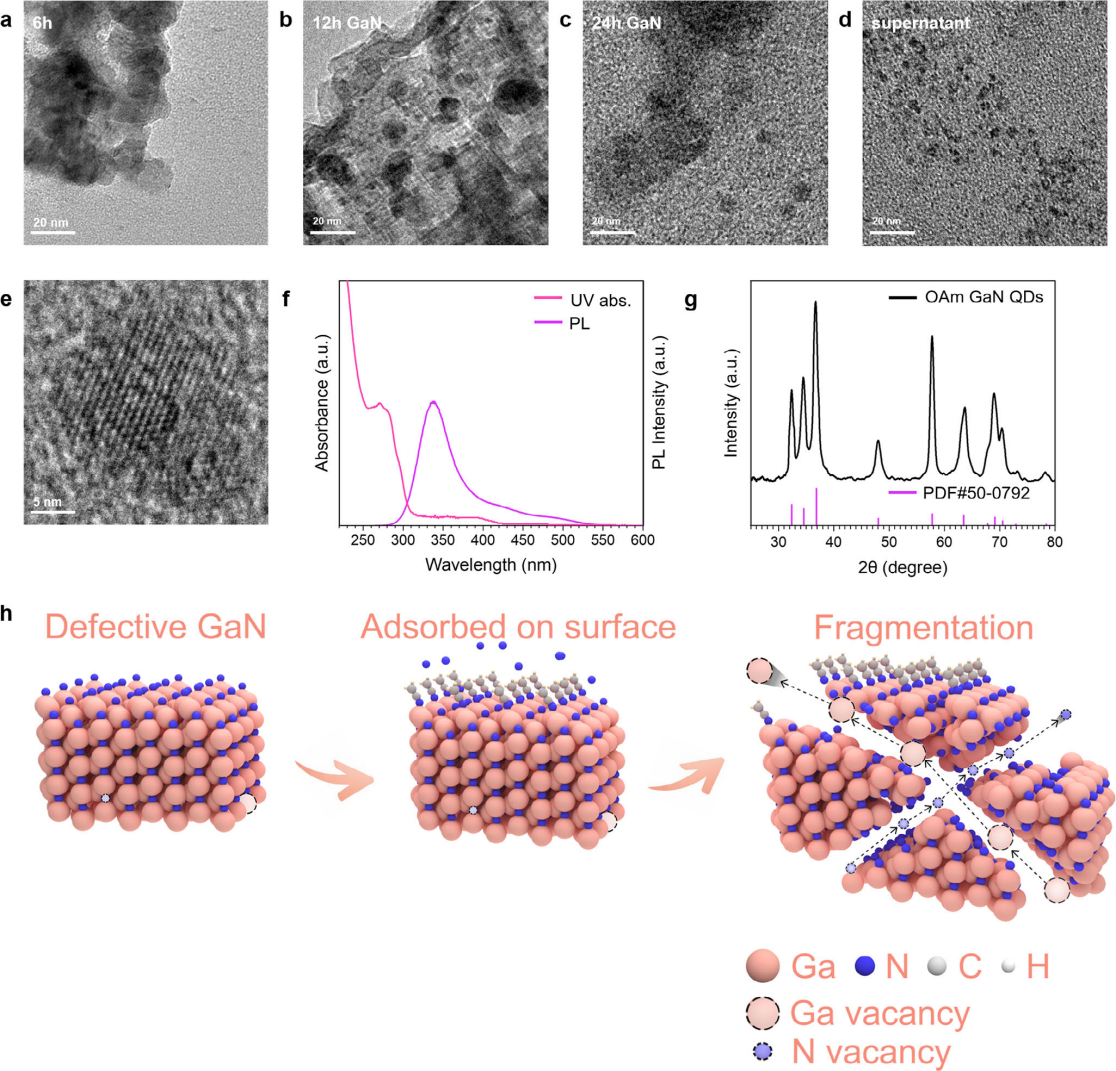
Synthesis and characterization of gallium nitride (GaN) quantum dots (QDs). (a–c) Time‐series transmission electron microscopy (TEM) images showing the progression of GaN fragmentation in oleylamine at 50°C, showing initial stage at 6 h (a), intermediate stage showing internal voids and channels at 12 h (b), and formation of discrete QDs at 24 h (c). (d) TEM image of GaN QDs isolated from the supernatant after centrifugation. (e) High‐resolution TEM (HRTEM) image of an individual GaN QD. (f) UV‐vis absorption (pink) and PL spectra (purple) of the GaN QD colloid under 270 nm excitation. (g) X‐ray diffraction (XRD) pattern of the synthesized GaN QDs, indexed to the wurtzite GaN phase (PDF#50‐0792). (h) Schematic of the proposed defect‐driven fragmentation mechanism.

Bulk GaN powder was dispersed in an organic medium, deoxygenated, and subsequently heated to 50°C to facilitate controlled chemical fragmentation and obtain colloidal QDs. The structural evolution during this process in oleylamine (OAm) was examined via transmission electron microscopy (TEM) of the reaction mixture aliquots at different time points (Figure 1a–c). The fragmentation is heterogeneous, with different regions of the bulk breaking down at varying rates, as captured in localized TEM snapshots. After 6 h (Figure 1a), the GaN particles remained largely intact, with minor grain formation at the surface. Fragmentation was more apparent by 12 h (Figure 1b) with the emergence of internal voids and channels, attributed to defect migration linking the particle's interior to its surface. These features likely contribute to evolving grain boundaries that destabilize the crystal framework and initiate subdivision into smaller domains. The fragmentation was largely complete after 24 h, yielding discrete, spherical GaN QDs released from the residual bulk framework. The colloidal QDs were then isolated from larger particles via centrifugation (Figure 1d; Figure S2). A high‐resolution TEM (HRTEM) image of an isolated GaN QD revealed a well‐defined crystalline structure (Figure 1e), with the periodic atomic arrangement resolved by inverse fast Fourier transform (IFFT) analysis (Figure S3). The particles were generally spherical with an average diameter of 4–11 nm, consistent with the localized and variable nature of fragmentation. The optical and structural properties of the fully fragmented QDs (168 h reaction time) confirmed the formation of high‐quality nanocrystals. A distinct peak at 270.5 nm, characteristic of GaN, was observed in the UV‐vis absorption spectrum. PL under 270 nm excitation revealed an emission peak at 338.8 nm (Figure 1f). This peak was significantly blue‐shifted from that of bulk GaN, evidencing quantum confinement. The FWHM of the emission profile was 48.8 nm, and the photoluminescence quantum yield (PLQY) was 7.6%. The asymmetric peak shape suggests multiple emissive pathways, including band‐edge and defect‐mediated transitions. X‐ray diffraction (XRD) (Figure 1g) patterns were well aligned with the standard peaks of wurtzite GaN. The crystallite size, calculated using the Scherrer equation, was 6–10 nm, consistent with the TEM results. We propose a defect‐driven fragmentation mechanism based on these observations (Figure 1h). We suggest that nitrogen‐containing organic species promote the formation of surface nitrogen vacancies (V_N_). This is thought to destabilize the GaN lattice and provide a thermodynamic driving force for the migration of internal defects toward the surface, subsequently contributing to the formation of polycrystalline domains and grain size reduction. As nanocrystals become isolated, the organic medium adsorbs onto their surfaces, passivating dangling bonds and stabilizing the colloidal GaN QDs.

### 2.2 Synthesis of GaN QDs in Alternative Nitrogen‐Containing Organic Species

**FIGURE 2:**
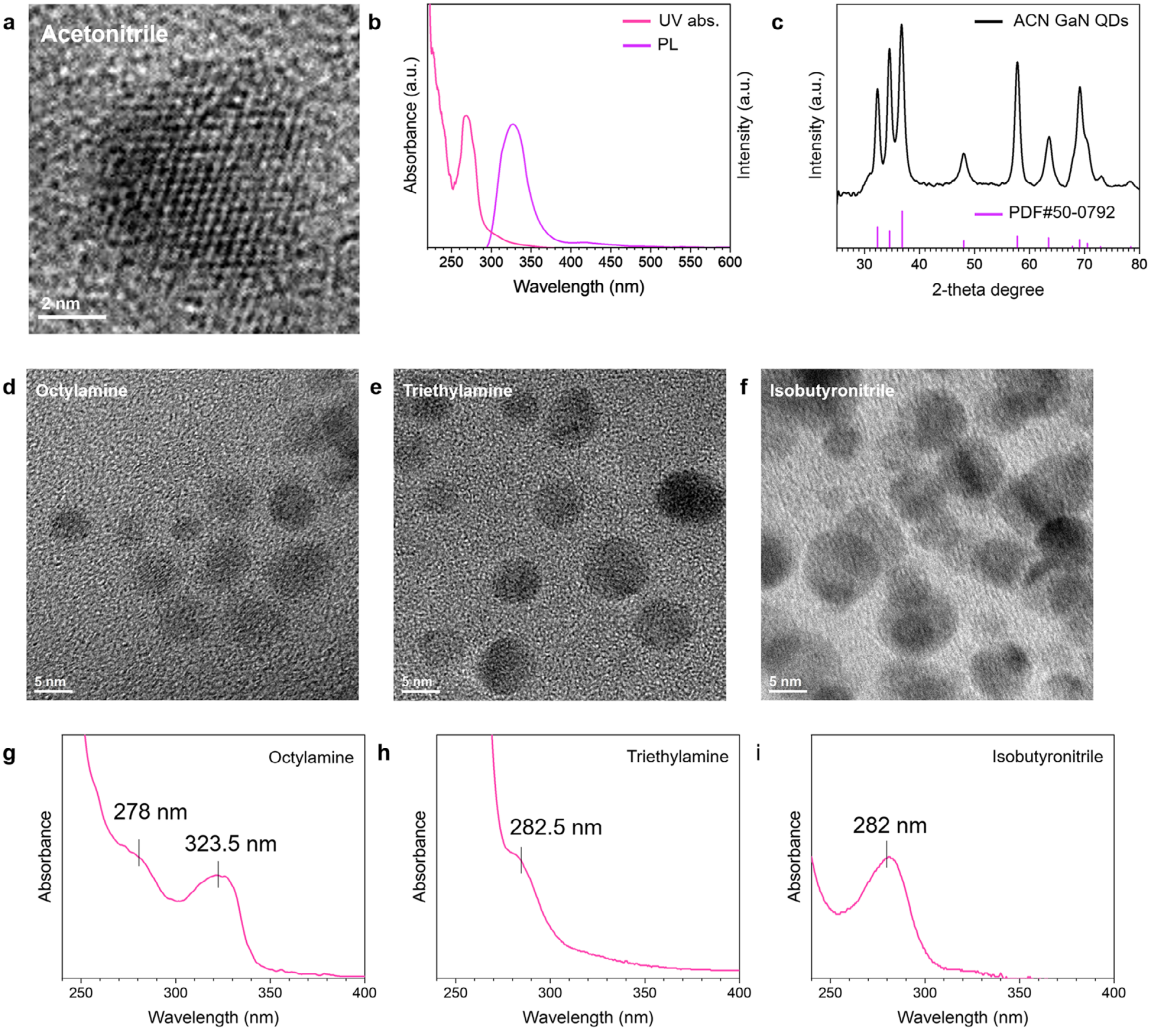
Fragmentation behavior in primary amines, tertiary amines, and nitriles. (a–c) Characterization of GaN QDs synthesized using acetonitrile (ACN), showing the HRTEM image (a), UV‐vis absorption (pink) and PL (purple) spectra under 270 nm excitation (b), and the XRD pattern indexed to the wurtzite GaN phase (c). (d–f) TEM images of GaN QDs synthesized in alternative nitrogen‐containing organic species, specifically octylamine (d), triethylamine (e), and butyronitrile (f). (g–i) Corresponding UV‐vis absorption spectra of GaN QDs synthesized with the same media as in (d–f), highlighting variations in spectral sharpness and absorption edge.

The scope and chemical requirements of fragmentation were investigated using a series of nitrogen‐containing organic solvents. By systematically lowering the reaction temperature of the OAm‐mediated synthesis, we first demonstrated that GaN QD formation proceeds successfully at temperatures as low as 50°C (Figure S3). Establishing this low‐temperature compatibility allowed us to expand the fragmentation media to include low‐boiling‐point solvents with minimal steric hindrance. Acetonitrile (ACN) was particularly effective. A representative TEM image of a nanoparticle synthesized in ACN revealed a single, well‐defined, crystalline QD (Figure 2a; Figure S4). The minimal steric hindrance of ACN facilitates its access to the bulk GaN surface, enabling the rapid synthesis of these high‐quality QDs. This kinetic requirement was further corroborated by control experiments using long‐chain dodecanenitrile (Figure S7), which resulted in severely arrested fragmentation. The optical properties indicated an improved monodispersity of the ensemble compared to the sample synthesized in OAm (Figure 2b). Compared to those of the product obtained using OAm, the absorption peak was sharper in the UV‐vis absorption spectrum, and the PL FWHM was reduced to 38.8 nm. The PLQY was enhanced to 15.3%. Transitioning the synthesized GaN QDs into electroluminescent devices presents specific surface‐chemistry challenges. The strongly coordinated organic ligands act as insulating barriers that hinder charge injection. Further, effective surface passivation is restricted by the wide bandgap of GaN. Achieving a Type‐I core/shell structure without introducing oxygen contamination necessitates the development of new synthesis methods for wider‐bandgap materials, such as AlN. Some gallium–amine coordination complexes can produce ultraviolet emission; therefore, the origin of the observed PL was ascertained via a control experiment. GaCl_3_ mixed with ACN under identical synthesis conditions exhibited no discernible PL (Figure S8). Emission from the solvents themselves was also excluded (Figure S9). Therefore, the strong emission in our samples was attributed to quantum‐confined GaN QDs. The crystallinity of the ACN‐derived QDs was verified via XRD: patterns indexed to the wurtzite GaN phase were observed (Figure 2c). Other nitrogen‐bearing media, including a primary amine (octylamine), a tertiary amine (triethylamine), and another nitrile (butyronitrile), were employed to assess the broader applicability of this fragmentation mechanism. Colloidal GaN QDs were formed in all cases (Figure 2d,f,h). The corresponding UV‐vis absorption (Figure 2e,g, i) indicated absorption onsets in a similar spectral region near 280 nm, confirming the successful formation of GaN QDs in each solvent. Thus, the defect‐driven fragmentation is a general process applicable to a range of nitrogen‐containing organic compounds, even though the spectral sharpness and resulting product uniformity depend on the specific solvent. The critical role of nitrogen for the chemical outcome of the reaction was confirmed via a control experiment using oleic acid, an oxygen‐containing organic compound. Although fragmentation was observed, optical analysis revealed an absorption peak at 230 nm and a PL spectrum characteristic of gallium oxide, not GaN, suggesting that the organic medium concurrently induces oxidation (Figure S10). Therefore, nitrogen‐containing species are necessary not only to initiate fragmentation but also to preserve the nitride phase. Incomplete inert gas purging resulted in the oxidation of the GaN surface and internal channels, leading to the formation of β‐Ga_2_O_3_ nanodomain byproducts, as confirmed via XRD (Figure S11). These results demonstrate that the proposed versatile yet chemically specific strategy for defect‐mediated GaN fragmentation requires a nitrogen‐based solvent and an oxygen‐free environment.

### 2.3 Defect Evolution During GaN Fragmentation

The changing defect landscape of GaN during fragmentation was examined by applying Gaussian deconvolution of the PL spectra to quantify the contributions from distinct emissive pathways, including quantum‐confined, band‐edge, and defect‐related transitions [24, 25, 26, 27, 28, 29].

**FIGURE 3:**
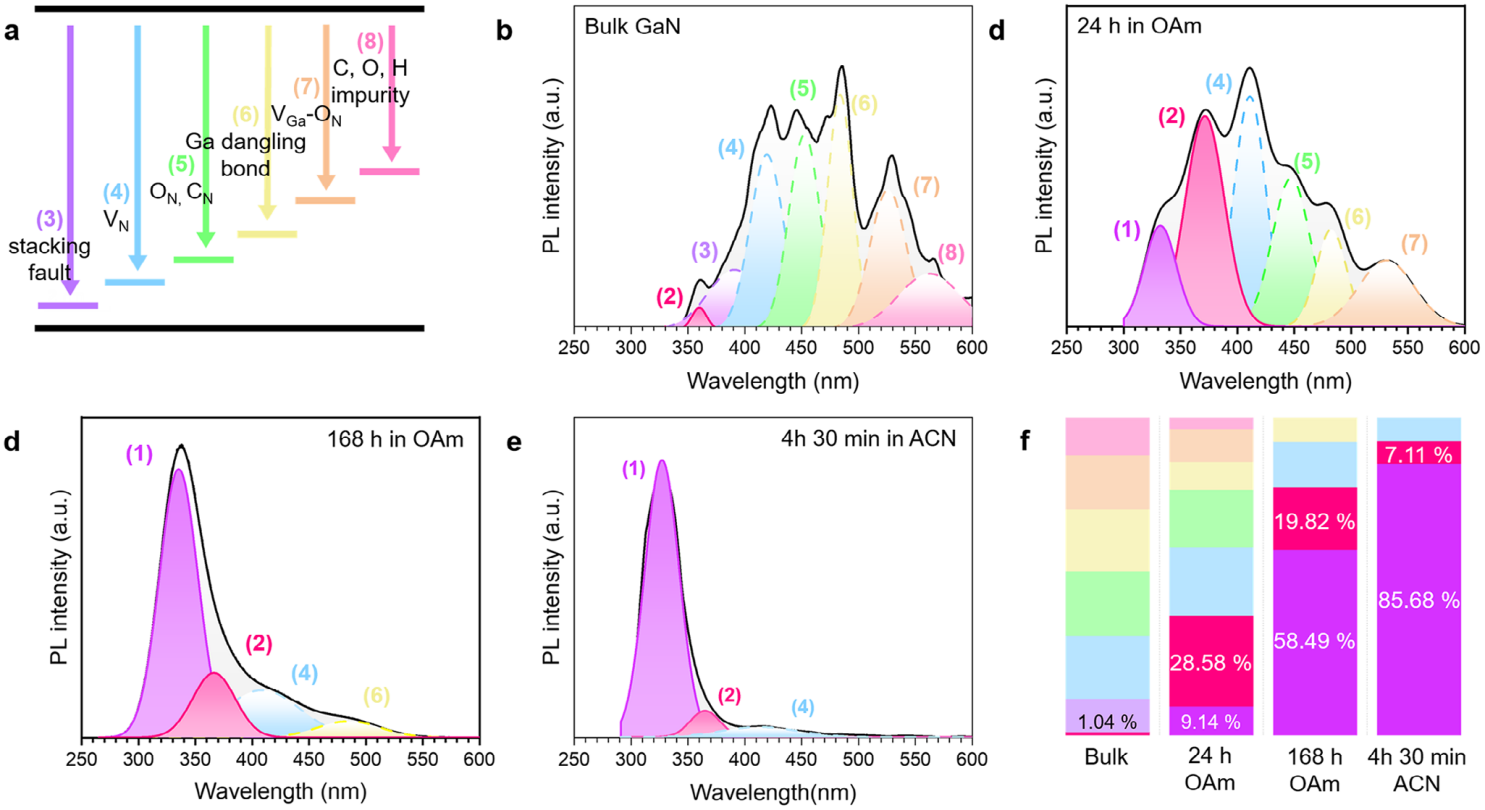
PL analysis and defect‐related luminescence changes. (a) Schematic of typical GaN defect states and their corresponding PL signatures. Each emission is color‐coded and indexed (1–8) to match the Gaussian components in panels (b–e). (b–d) Gaussian deconvolution of PL spectra showing the evolution of defect states during GaN fragmentation in oleylamine for the pristine polycrystalline GaN powder (b), after 24 h of fragmentation (c), and after 168 h (d). (e) Gaussian deconvolution of the PL spectrum from GaN QDs synthesized in acetonitrile after only 4 h 30 min of reaction. (f) Relative area fractions of each deconvoluted emission component over fragmentation time in oleylamine and under rapid synthesis in acetonitrile.

The specific assignments for eight representative emission bands in GaN, based on the literature, are summarized in a schematic (Figure 3a) [30, 31, 32, 33, 34, 35, 36, 37]. The PL spectrum of the pristine, as‐received bulk GaN was dominated by broad, defect‐related luminescence, with negligible contributions from band‐edge emission (1.04%), indicating a high initial density of structural and impurity‐related defects (Figure 3b). The defect states were progressively eliminated upon fragmentation in OAm. After 24 h (Figure 3c), the overall defect emission decreased considerably, and the band‐edge emission component increased to 28.58%. A new peak, attributed to quantum confinement in nanocrystals smaller than the GaN exciton Bohr radius, emerged at 335.3 nm [38]. The 390 nm emission band—associated with strain and stacking faults—was eliminated, indicating that the fragmentation process resolves pre‐existing structural distortions and relieves internal crystal stress. By 168 h (Figure 3d), the PL spectrum was dominated by the quantum‐confined component (58.49% of the total emission intensity), signifying the conversion of the bulk material into a population of high‐quality QDs with a low residual defect density. The emergence of defect emission only after hydrofluoric acid etching (Figure S12) verifies the high quality of the original QDs. A comparative analysis using ACN as the solvent revealed a significantly accelerated fragmentation process. The quantum‐confined component constituted 85.68% of the total emission after only 4 h 30 min of reaction (Figure 3e), and deep‐level defect emissions were suppressed to 7.21%. The emergence of emissions related to oxygen impurities and gallium vacancies only after intentional oxidation (Figure S13) confirms the high crystalline quality of the as‐synthesized QDs. Thus, ACN facilitates a more rapid and complete defect extraction compared to OAm. The overall trend is summarized in Figure 3f, which shows the relative area of each emission component as a function of reaction time. The clear inverse correlation—a progressive decrease in all defect‐related emissions with a concurrent increase in the quantum‐confined emission—serves as a spectroscopic fingerprint for the defect‐driven fragmentation mechanism. Furthermore, the decrease in signal intensity observed in the electron paramagnetic resonance spectra of the GaN QDs compared to the bulk GaN (Figure S14) indicates reduction in internal defects during the fragmentation process, corroborating the proposed mechanism. This combined spectroscopic evidence supports a model in which native defects are not merely passivated but are physically driven to the surface and removed from the crystal lattice as the bulk material subdivides into discrete GaN QDs.

### 2.4 Density Functional Theory (DFT) Calculations

The relationship between defects and GaN fragmentation was further investigated via first‐principles calculations. The gradual reduction in defects with GaN decomposition suggests a strong correlation between defect formation and fragmentation. Based on the XRD results (Table S1), the $\left(10\bar{1}1\right)$ surface was identified as a dominant exposed facet of the bulk GaN powder and was therefore selected as a representative surface for the analyses. N‐dimer reconstruction readily occurred on this surface (Figure S15) [39].

**FIGURE 4:**
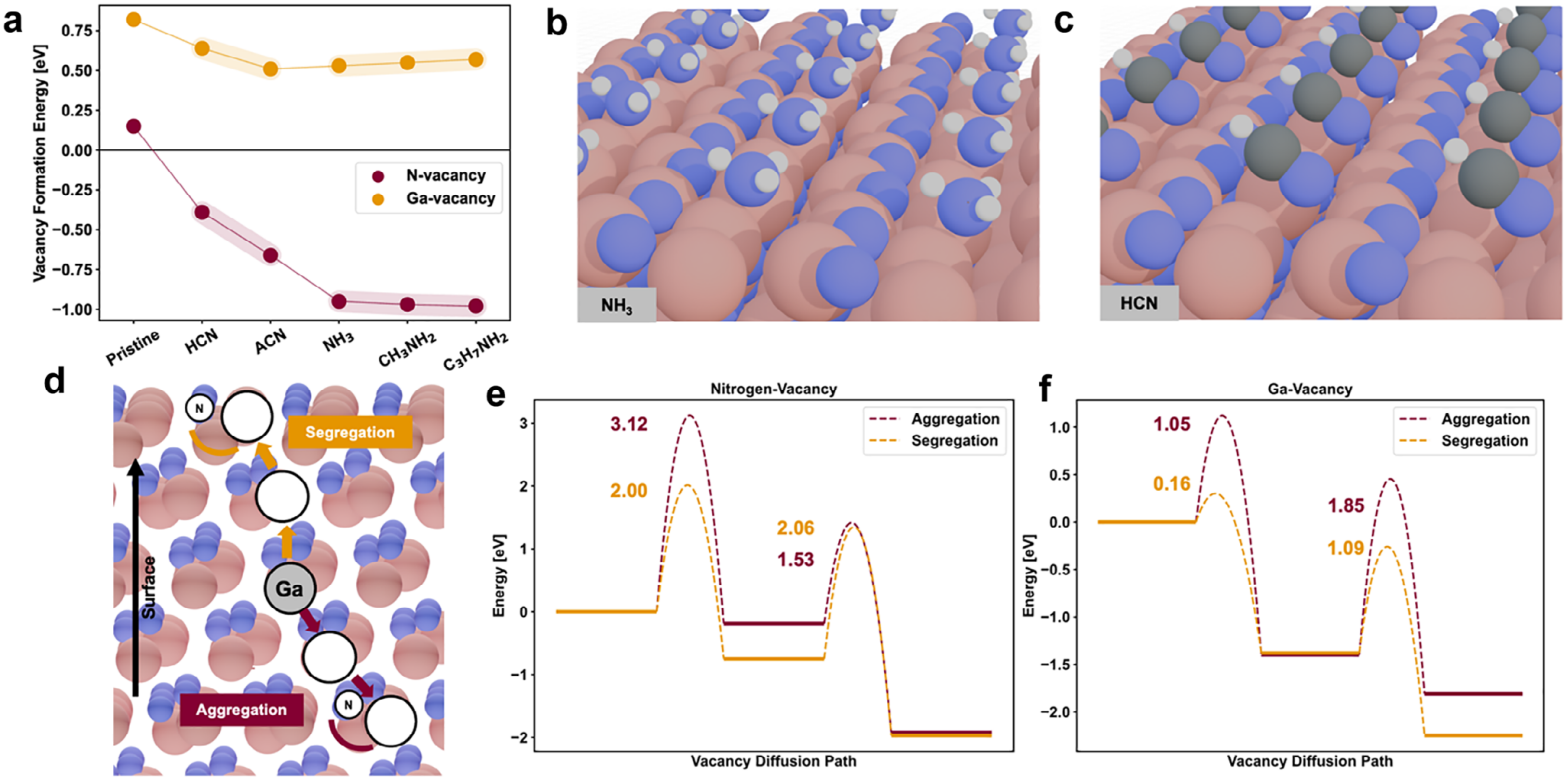
Vacancy formation energies and kinetic barrier analysis. (a) Formation energies for V_N_ and V_Ga_ on the pristine GaN (10$\bar{1}$1) surface and with various adsorbates. (b,c) Optimized structures of GaN surfaces containing an V_N_ with adsorbed NH_3_ (b) and HCN (c), respectively. (d) Schematic of vacancy migration mechanisms: segregation, wherein bulk vacancies diffuse toward a newly formed surface vacancy, and aggregation, wherein bulk vacancies migrate into existing defects. (e,f) Calculated kinetic barriers for V_N_ (e) and V_Ga_ (f) migration via segregation and aggregation. Atom colors: pink (Ga), blue (N), gray (C), and white (H).

The effect of nitrogen‐containing organic solvents on defect formation was evaluated using both the representative adsorbates (NH_3_ and HCN) and organic molecules (acetonitrile, methylamine, and propylamine). Upon their adsorption, the formation energy of V_N_ decreases markedly relative to the pristine surface and becomes exothermic, whereas V_Ga_ formation, although also lowered, remains endothermic under all conditions (Figure 4a). This contrast holds consistently across all adsorbates, with the V_N_ values for the primary amines converging as the alkyl chain length increases. The robustness of this trend—exothermic V_N_ versus endothermic V_Ga_, independent of the specific adsorbate—indicates that it is the general N‐coordinating character of these organics, rather than any particular molecular structure, that promotes nitrogen vacancy formation. The corresponding adsorption configurations of the organic molecules are provided in Figure S16. The stabilization of V_N_ is attributed to the N‐terminated functional groups, which effectively passivate defect sites and facilitate vacancy formation, as illustrated by the representative NH_3_ and HCN adsorption geometries (Figure 4b,c). These theoretical findings are consistent with X‐ray photoelectron spectroscopy (XPS) data, which show that the Ga^2+^ peak is absent in the pristine state, but appears when nitrogen‐containing solvents are present. Fragmentation proceeds via V_N_ generation rather than V_Ga_ formation owing to the observed thermodynamic difference: only V_N_ formation is exothermic. To further account for the solvent environment, we additionally performed implicit solvation calculation for the V_N_ formation step, which preserved the exothermic trend (Figure S17), indicating that the conclusions are robust to dielectric screening by the surrounding medium.

The formation of surface V_N_ dynamically alters the overall vacancy concentration, leading to vacancy reconstruction via segregation or aggregation (Figure 4d). Segregation involves bulk vacancies diffusing to the surface because of newly formed surface vacancies. Aggregation involves bulk vacancies diffusing into existing bulk defects. The tendency of Ga and N vacancies to cluster together drives these mechanisms (Figure S18) [40].

The diffusion behavior of these vacancies, facilitated by the newly introduced vacancies, was studied via kinetic barrier analysis (Figure S19; Figure S20). For bulk V_Ga_, the rate‐determining step was 1.09 eV for segregation and 1.85 eV for aggregation (Figure 4e). Therefore, V_Ga_ preferentially diffuses to the surface rather than remaining in the bulk for aggregation. V_Ga_ at the surface might further promote rearrangement by clustering with other vacancies, thereby creating sites that induce the upward segregation of V_N_ (2.06 eV vs. 3.12 eV) (Figure 4f). These results suggest sequential defect migration: surface V_N_ formation initiates the segregation of bulk V_Ga_, which, in turn, facilitates further V_N_ segregation. This proposed chain of vacancy migration provides a mechanism for GaN fragmentation (Figure 1h). The preferential segregation of both V_Ga_ and V_N_ to the surface leads to vacancy accumulation, which gradually disrupts lattice continuity and generates localized structural instability. Such vacancy‐enriched domains reduce bond cleavage energy and facilitate polycrystalline region formation. These findings align with previous studies reporting that defect diffusion pathways and the associated redistribution of vacancies are critical for initiating lattice weakening and facilitating fragmentation [41].

### 2.5 Determination of the Fragmentation Pathway via Residual GaN Framework Analysis

**FIGURE 5:**
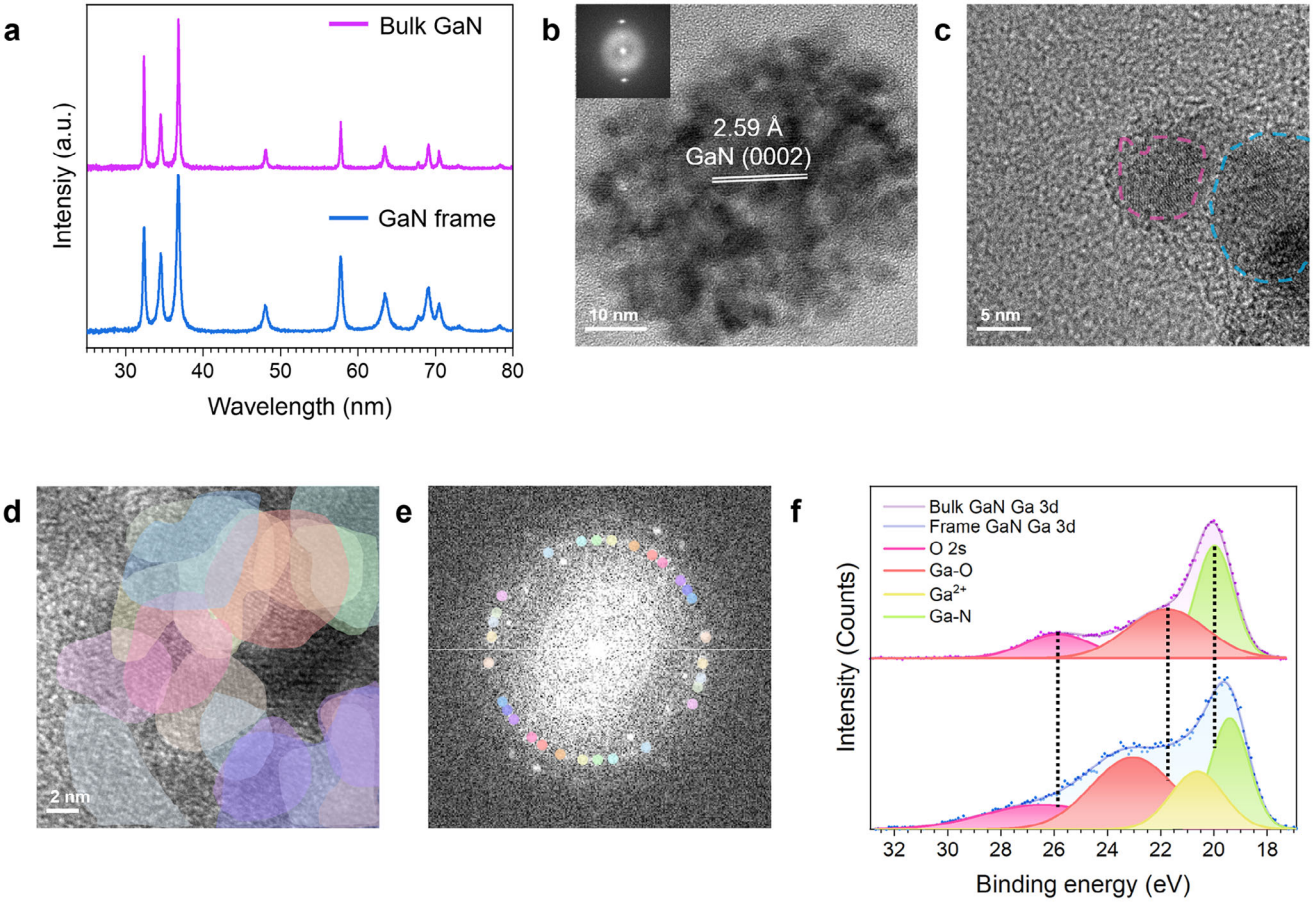
Structural and chemical analysis of the residual GaN framework. (a) XRD patterns of pristine bulk GaN (top) and the residual GaN framework after 4 h 30 min fragmentation in ACN (bottom). (b) TEM image and the corresponding fast Fourier transform (FFT) of a largely single‐crystalline region within the GaN framework. (c) TEM image showing channel formation and the emergence of discrete GaN QDs (false‐colored purple) from the parent matrix (false‐colored blue). (d) Overlay of a TEM image from a polycrystalline region with color‐coded inverse fast Fourier transform (IFFT) images derived from individual diffraction spots, revealing extensive crystallite subdivision. (e) Corresponding FFT of the region in panel d; colored circles indicate the spots used for the IFFT reconstructions. (f) Ga 3d X‐ray photoelectron spectroscopy (XPS) spectra of pristine bulk GaN (top) and the residual GaN framework (bottom).

The mechanism of QD formation via ACN‐mediated fragmentation was investigated via structural and chemical analyses of the residual bulk material (hereafter, GaN framework) [42, 43]. Because XRD is a bulk characterization technique independent of electron beam exposure, it confirms that the observed structural changes occur macroscopically rather than as localized artifacts of thin‐specimen preparation. The XRD peaks for the post‐fragmentation GaN framework were considerably broader than those for the bulk GaN precursor, indicating grain size reduction (Figure 5a). Scherrer analysis revealed a crystallite size reduction of 43.5–71.4%, with the extent of reduction varying by crystallographic orientation. This bulk‐scale verification indicates that fragmentation proceeds via internal grain subdivision (Table S1). The nanoscale heterogeneity of this fragmentation process was further elucidated via TEM imaging of the GaN framework. Some regions corresponding to an early stage of fragmentation remained largely single‐crystalline, exhibiting a coherent GaN (0002) lattice with minor distortions (Figure 5b; Figure S21). In contrast, advanced fragmentation stages were characterized by pronounced channel formation and the clear emergence of discrete GaN QDs from the parent matrix (Figure 5c; Figure S22). The extensive grain subdivision was visualized via IFFT analysis of a polycrystalline region of the framework (Figure 5d; Figure S23). At least 15 discrete crystallites were resolved within a single particle by masking individual diffraction spots in the corresponding fast Fourier transform (FFT) (Figure 5e) and overlaying the color‐coded IFFT reconstructions; these results confirm the progressive formation of polycrystalline domains during fragmentation. Chemical changes were probed via XPS survey scans (Figure S24) and high‐resolution Ga 3d XPS spectra of the pristine bulk GaN and residual GaN framework (Figure 5f). The bulk GaN spectrum was deconvoluted into three peaks, whereas a fourth component at 20.62 eV was required for the GaN framework spectrum. This new feature is attributed to Ga^2+^ states resulting from the formation of surface nitrogen vacancies during fragmentation [44]. XPS analysis after just 15 min of reaction (Figure S25) reveals the emergence of these Ga^2+^ states before shifts in Ga–N or Ga–O bonding occur. This temporal sequence supports our hypothesis that surface destabilization by the nitrogen‐containing solvent initiates defect migration and subsequent grain subdivision. Conversely, a control experiment using 1‐octadecene—a non‐coordinating solvent—produced neither a Ga^2^^+^ peak nor any reduction in crystallite size (Figure S26), confirming that chemical surface destabilization is required. Finally, in the fully fragmented framework, concurrent shifts in Ga–N and Ga–O bonding indicate that the extensively destabilized surface becomes highly susceptible to oxidation upon handling, as further evidenced by Ga 2p (Figure S27) and N 1s (Figure S28) spectra and their corresponding deconvolution parameters (Table S2). These results provide supporting experimental observations for nitrogen vacancy formation and structural subdivision, which is consistent with the proposed defect‐driven fragmentation mechanism.

**FIGURE 6:**
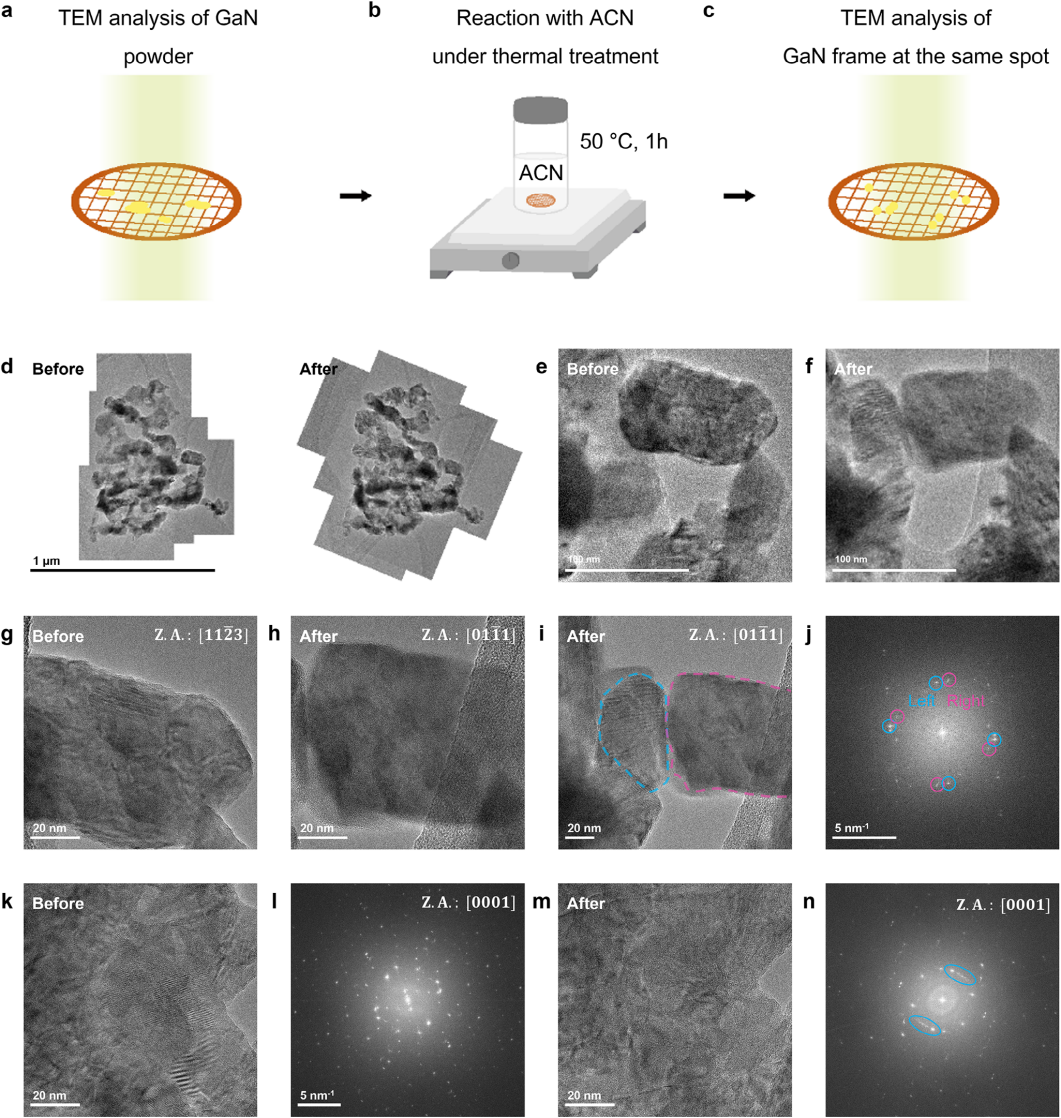
ILTEM analysis of GaN. (a–c) Schematic of the identical‐location transmission electron microscopy (ILTEM) procedure, showing low‐dose TEM imaging of GaN powder (a), ACN treatment on the TEM grid (b) and post‐treatment imaging (c). (d) Low‐magnification TEM images of GaN powder before and after ACN treatment. (e,f) TEM images of GaN powder at the same magnification before (e) and after (f) treatment, showing a cracked particle. (g,h) HRTEM images of the cracked GaN particle before (g) and after (h) treatment. (i,j) Phase analysis of the cracked GaN particle after treatment, showing the HRTEM image (i) and its corresponding FFT (j), with peaks from the left (blue) and right (purple) regions indicated. (k,l) Phase analysis of a GaN particle with a stacking fault before treatment, showing the HRTEM image (k) and its corresponding FFT (l). (m,n) Phase analysis of the particle after treatment, showing the HRTEM image (m) and its corresponding FFT (n), with peaks from the stacking fault (blue) indicated.

The fragmentation process at the nanoscale was verified via identical‐location transmission electron microscopy (ILTEM). Conventional TEM analysis captures different particles at each time point, and hence, resolving the sequential changes within an individual crystal is challenging. In particular, distinguishing between polycrystalline morphology pre‐existing as a grain boundary and due to fragmentation is difficult. ILTEM enables site‐specific tracking of morphological and structural changes within the same particle by fixing a region of the crystal and imaging it before and after chemical treatment. Thus, ILTEM tracks the evolution of a single‐crystalline domain into a polycrystalline fragment, providing real‐space observations of grain subdivision and internal defect evolution. For ILTEM analysis, GaN powder dispersed in ethanol was drop‐cast onto a TEM grid. HRTEM imaging was first performed to minimize electron beam‐induced damage (Figure 6a). The grid was then immersed in ACN and mildly heated at 50°C for 1 h to induce chemical interaction (Figure 6b). HRTEM imaging was subsequently conducted at the same location to monitor structural changes (Figure 6c). ILTEM revealed local structural deformation in the GaN particles, such as cracking and defect formation, following ACN treatment. Low‐magnification TEM images (Figure 6d) indicated that the overall particle morphology was mostly intact. However, higher magnification images (Figure 6e,f) indicated a noticeable crack, with the particle splitting into two distinct domains after treatment (Figure 6f). This cracking, combined with drying‐induced stress or ACN‐induced rotation, may cause particle reorientation, as suggested by a change in the zone axis before and after treatment (Figure 6g,h; Figure S29). Further high‐resolution analysis of the cracked particle revealed twisting, with the left and right domains exhibiting a noticeable angular tilt relative to each other (Figure 6i,j; Figure S30). The reaction appeared to induce structural defects, particularly at grain boundaries. A polycrystalline GaN particle (Figure 6k), identified through FFT analysis (Figure 6l), exhibited stacking faults and other defects at the grain boundaries after ACN exposure (Figure 6m). Because chemical reactions often proceed more rapidly at grain boundaries than in the bulk, these boundaries are the probable defect formation sites. ILTEM confirmed that ACN treatment induces deformation in GaN particles, observable at the single‐particle level. This deformation is likely promoted by ACN adsorption at nitrogen vacancy sites on the GaN surface, facilitating local structural changes that may ultimately contribute to GaN QD formation.

## 3 Conclusion

We demonstrated a low‐temperature, solution‐based route to wurtzite GaN colloidal QDs via the chemical fragmentation of bulk GaN powder. This approach circumvents the extreme temperature and pressure conditions typical of GaN synthesis by leveraging a defect‐driven fragmentation mechanism. The organic solvent induces nitrogen defects on the GaN surface, which act as sinks that promote internal vacancy migration, ultimately triggering the mechanical scission of the bulk crystal into nanoscale domains. This top‐down pathway yields GaN QDs with high crystallinity, size‐dependent quantum confinement, and distinct band‐edge emission. The optimized synthesis achieves an FWHM of 38.8 nm and a PLQY of 15.3%. Furthermore, QD properties can be tuned via solvent selection; the distinct chemical environments provided by solvents such as OAm and ACN dictate the particle size, luminescence, and surface defect passivation. This non‐conventional, scalable strategy provides a promising approach for addressing key challenges in the defect engineering and surface chemistry of III‐nitride nanocrystals. Our results can help accelerate the development of GaN‐based quantum materials for next‐generation applications in nanophotonics and solid‐state lighting.

## Author Contributions

Y.J. conceived and designed the study, conducted the synthesis of GaN quantum dots, and performed all experimental characterizations including TEM, XRD, UV‐vis absorption, photoluminescence (PL) measurements, Gaussian deconvolution of PL spectra, XPS measurements, and analysis of IFFT images. K.K. carried out the DFT calculations and simulations related to the fragmentation. H.B. contributed to obtaining the identical‐location TEM (ILTEM) images. J.P. supervised the experimental work and provided project guidance. J.H. supervised the computational component of the study. Y.J. and K.K. co‐wrote the manuscript with input from all authors. All authors contributed to the discussion and revision of the manuscript.

## Conflicts of Interest

The authors declare no conflicts of interest.

## Supporting information




**Supporting File**: smtd70956‐sup‐0001‐SuppMat.docx.

## Data Availability

All data supporting the findings of this study are available within the article and its Supplementary Information files. Additional raw data (including PL spectra, TEM images, XRD patterns, and Gaussian deconvolution results) are available from the corresponding author upon reasonable request.
