# Supplementary material for: Top‐Down Synthesis of Wurtzite Gallium Nitride Colloidal Quantum Dots via Defect‐Driven Fragmentation
Source: Small Methods. 2026 Aug 19;10(18):e70956. doi: 10.1002/smtd.70956 (PMC13599707; doi:10.1002/smtd.70956)
Supplement: Supplementary file 1 [file SMTD-10-e70956-s001.docx]

Supporting Information

**Top-Down Synthesis of Wurtzite Gallium Nitride Colloidal Quantum Dots via Defect-Driven Fragmentation**

Yangpil Jang^1†^, Kwangwook Kim^2†^, Hayeon Baek^3^, Dongjun Kim^3^, Jungwon Park^3^, Jeong Woo Han^2^* and Jongnam Park^1,4^*

Methods

Chemicals

Gallium nitride (GaN, 99.9% trace metals basis), oleic acid (technical grade, 90%), 1-octadecene (technical grade, 90%), OAm (technical grade, 70%), ACN (≥99.5%, ACS reagent), octylamine (≥99%), triethylamine (≥99%), and isobutyronitrile (99%) were purchased from Sigma-Aldrich and used as received without further purification.

Synthesis of GaN QDs in OAm

In a typical synthesis, 0.12 mmol of GaN powder was added to a round-bottom flask containing 57.44 mmol of OAm. The mixture was vigorously stirred and degassed at 110 °C under vacuum for 2 h to remove dissolved gases and trace oxygen. After purging with argon, the temperature was increased to 170 °C, and the reaction proceeded under constant stirring. After the desired reaction time(6, 24, 168 h), the mixture was cooled and centrifuged at 9000 rpm for 15 min to separate unreacted bulk GaN. The supernatant, containing colloidally dispersed GaN QDs, was collected for further characterization.

Synthesis of GaN QDs in ACN and other organic species

GaN QDs were also prepared using various nitrogen-based solvents to evaluate the universality of the fragmentation-driven synthesis. Specifically, 0.12 mmol of GaN powder was dispersed in 57.44 mmol of the target solvent (ACN, octylamine, triethylamine, or isobutyronitrile) in a sealed vial. Acetonitrile is toxic and flammable; all reactions were performed in a fume hood. The solution was purged with argon gas for 3 h to remove residual oxygen. The sealed reaction mixture was then vigorously stirred at 50 °C for 4 h 30 min. The mixture was centrifuged after the reaction to remove unfragmented GaN, and the supernatant containing GaN QDs was collected for analysis. To confirm the scalability of this top-down approach, a 100-fold scaled-up synthesis was concurrently performed. In this scaled reaction, 12.0 mmol of GaN powder was dispersed in 5.74 mol of ACN under identical degassing and reaction conditions, yielding optically and structurally consistent GaN QDs.

Characterization

Absorption spectra were recorded using a Shimadzu UV-1800 UV-vis spectrophotometer, and PL spectra were acquired using an Agilent Cary Eclipse fluorescence spectrophotometer. The inner wall of the integrating sphere was coated with a layer of polytetrafluoroethylene, which is highly reflective in the UV spectral range down to 200 nm. TEM images were acquired using a JEOL JEM-2100 at an acceleration voltage of 200 kV. A Rigaku D/MAX2500V/PC X-ray diffractometer operated at 40 kV/200 mA using the Cu Kα line (λ = 1.5405 Å) was used for XRD. X-ray photoelectron spectroscopy measurements were acquired using a Thermo Fisher K-Alpha spectrometer. The Frame GaN sample was dried in a vacuum oven for 12 hours to remove residual organic matter from the surface.

Changes in the structures of individual GaN particles before and after ACN treatment were visualized via ILTEM. This ILTEM protocol allowed direct, side-by-side visualization of lattice fringes, clearly revealing GaN particle deformation and surface reconstruction following ACN treatment. The specimens were prepared as follows. GaN powder was dispersed in anhydrous ethanol (0.001 g mL⁻¹) and ultrasonicated for 5 min to ensure a homogeneous suspension. A 3 µL aliquot of the dispersion was then deposited onto lacey carbon-coated Cu TEM grids (200 mesh). For ILTEM mapping, low-magnification overview images (×60,000) were first recorded to register landmark particles, and high-resolution images of the same regions were acquired at ×200,000 and ×300,000 (0.054 and 0.036 nm pixel⁻¹, respectively). The grid was then immersed in neat ACN and gently heated at 50 °C for 1 h to induce chemical interaction. After treatment, the identical regions were relocated using the recorded stage coordinates, and both low- and high-magnification images were reacquired. All micrographs were collected using a JEOL JEM-ARM200F operated at 200 kV and equipped with a cold field-emission gun, CEOS objective-lens spherical-aberration corrector, and scintillator-based CMOS camera (OneView, Gatan). This IL-HRTEM workflow was used to obtain one-to-one visualization of lattice fringes from identical GaN crystallites before and after ACN exposure, offering detailed insight into particle deformation and surface reconstruction.

Computational details

DFT calculations were performed using the Vienna Ab-initio Simulation Package. The generalized gradient approximation with the Perdew–Burke–Ernzerhof exchange–correlation functional was employed. The plane-wave cutoff energy was set to 400 eV. Electron smearing was treated using the Gaussian smearing method with a width of 0.05 eV, suitable for insulating systems. Electronic relaxation was considered until the energy difference between successive steps was less than 1 × 10⁻⁶ eV. The ionic relaxation was performed using a conjugate gradient algorithm, with a maximum of 800 ionic steps and a force convergence criterion of 0.05 eV/Å. The Brillouin zone was sampled with a 5 × 5 × 1 Monkhorst–Pack *k*-point mesh.

The GaN (10$\overline{\text{1}}$1) surface was modeled using a slab consisting of 9 atomic layers with a vacuum thickness of 20 Å to avoid the interactions between periodic images. A 2 × 2 lateral supercell was used to minimize artificial interactions between defects. During structural relaxation, the bottom 3 layers were fixed, while the remaining atoms were fully relaxed to simulate the surface environment.

The vacancy formation energies ($\text{E}_{\text{vac}}$) of nitrogen (N) and gallium (Ga) were calculated using equation (1):

$\text{E}_{\text{vac}}\text{= }\text{E}_{\text{total}}^{\text{defect}}\text{- }\text{E}_{\text{total}}^{\text{pris}}\text{+ μX}$	(1)

where $\text{E}_{\text{total}}^{\text{defect}}$ and $\text{E}_{\text{total}}^{\text{pris}}$ denote the total energies of the defective and pristine systems, respectively, and μX represents the chemical potential of the removed atom (N or Ga) referenced to its bulk phase. μGa was derived from metallic gallium in its equilibrium crystal structure; μN was computed using molecular nitrogen (N_2_) in the gas phase.

The adsorption energies (E_ads_) for NH_3_ and HCN molecules on the surface were calculated using equation (2):

$\text{E}_{\text{ads}}\text{= }\text{E}_{\text{total}}^{\text{slab+adsorbate}}\text{- }\text{E}_{\text{slab}}\text{- }\text{E}_{\text{adsorbate}}$	(2)

where $\text{E}_{\text{total}}^{\text{slab+adsorbate}}\text{, }\text{E}_{\text{slab}}\text{, }\text{and}\text{ }\text{E}_{\text{adsorbate}}$ denote the total energies of the adsorbed system, clean slab, and isolated adsorbate molecule, respectively.

The energy barrier for vacancy diffusion was computed using the climbing-image nudged elastic band method. Three intermediate images were interpolated between initial and final vacancy configurations, and atomic positions were relaxed until forces perpendicular to the reaction path fell below 0.05 eV/Å.

**Figure S1.** TEM characterization of the fragmentation products. **a, b** Transmission electron microscopy (TEM) images showing the residual GaN framework and the synthesized GaN QDs after 24 h of fragmentation. **c** High-magnification TEM image detailing the nanoscale morphology of the fragmented particles.

**Figure S2.** **a**,**b** High-resolution TEM (HRTEM) images of fragmented GaN nanoparticles after 24 h of reaction and **c**,**d** the corresponding fast Fourier transform (FFT). Although the selected particles appear to be single-crystalline in FFT, internal lattice defects are clearly visible in the HRTEM image. The photoluminescence (PL) spectrum of this sample exhibits significant defect-related emission, consistent with the presence of intrinsic lattice imperfections.

**Figure S3.** UV-vis absorption spectrum of GaN QDs synthesized in oleylamine at 50 °C.

**Figure S4.** Crystallographic analysis of GaN QDs synthesized in oleylamine. **a**, HRTEM image. **b–d**, inverse fast fourier transform (IFFT) images corresponding to individual FFT diffraction spots. **e**, Composite HRTEM image enhanced by overlaying the IFFT components. **f–h**, corresponding FFT regions.

**Figure S5.** Morphology and size distribution of the synthesized GaN QDs. **a** TEM image. **b** Corresponding particle size distribution histogram.

**Figure S6.** Low-magnification TEM image of GaN QDs synthesized in acetonitrile.

**Figure S7.** UV-vis absorption spectrum of GaN QDs synthesized in dodecanenitrile.

**Figure S8.** PL spectrum of the GaCl_3_–acetonitrile mixture under synthesis conditions. No distinct PL features were observed above background noise.

**Figure S9.** PL spectra of nitrogen-bearing organic solvents used in synthesis. **a**, Acetonitrile. **b**, Oleylamine. Excitation wavelength: 270 nm.

**Figure S10.** Optical properties of GaN QDs synthesized in oleic acid. **a**, UV-vis absorption spectrum. The GaN peak at 270 nm is retained, and the Ga_2_O_3_ absorption near 230 nm is enhanced. **b**, PL spectrum under 230 nm excitation.

**Figure S11.** X-ray diffraction (XRD) analysis of GaN powder dispersed in acetonitrile under air atmosphere. The sample shows distinct peaks corresponding to β-Ga_2_O_3_, indicating surface oxidation due to insufficient oxygen exclusion during synthesis.

**Figure S12.** PL spectrum of GaN quantum dots (QDs) synthesized in oleylamine following hydrofluoric acid treatment. Nitrogen vacancy related defect emission is notably enhanced post-treatment.

**Figure S13.** PL spectral evolution of GaN QDs synthesized in acetonitrile after oxidation. Following heating at 70 °C in air for 4 h 30 min, emissions associated with oxygen impurity(O_N_), gallium vacancy(V_Ga_) , and V_Ga_–O_N_ defects are enhanced.

**Figure S14.** Electron paramagnetic resonance spectroscopy. **a** EPR spectrum of the pristine bulk GaN. **b, c** EPR spectra of the synthesized GaN QDs.

**Figure S15.** Schematic of nitrogen-dimer (N_2_) surface reconstruction on the GaN (10$\overline{\text{1}}$1) plane. Surface relaxation results in the formation of an N–N dimer (highlighted in red), illustrating the tendency of surface nitrogen atoms for reconstruction via dimerization.

**Figure S16.** Density functional theory (DFT)- optimized adsorption configurations of the actual solvent molecules on the GaN $(10\overline{\text{1}}1)$ surface. (**a–c**) Side views and (**d–f**) top views of (**a, d**) acetonitrile (CH₃CN), (**b, e**) methylamine (CH₃NH₂), and (**c, f**) propylamine (C₃H₇NH₂) adsorbed on the surface.

**Figure S17.** Comparison of vacancy formation energies on the GaN $(10\overline{\text{1}}1)$ surface calculated in vacuum and with implicit solvation (VASPsol).

**Figure S18.** Density functional theory (DFT)-calculated formation energies of Ga and N vacancy pairs in bulk GaN. Vacancy locations are marked by red (V_Ga_) and yellow (V_N_) circles. The total energy is the lowest for Case 1, in which Ga and N vacancies are the closest, indicating a thermodynamic preference for vacancy clustering.

**Figure S19.** Schematic of two V_N_ migration pathways: segregation (top) and aggregation (bottom). In segregation, V_N_ migrates to the surface, increasing the vacancy concentration near the surface. In aggregation, V_N_ moves toward existing V_Ga_ sites in the bulk, leading to clustering.

 **Figure S20.** Schematic of two V_Ga_ migration pathways: segregation (top) and aggregation (bottom). In segregation, V_Ga_ migrates toward the surface, enriching surface vacancy density. In aggregation, V_Ga_ moves toward a V_N_ site, resulting in defect coalescence.

**Figure S21.** Magnified FFT image of Figure 5b, highlighting grain distortion.

**Figure S22.** Microstructural analysis of the GaN framework. **a**, TEM image showing multiple fragmentation channels. **b**, Corresponding FFT, confirming polycrystallinity post-channel formation.

**Figure S23.** Unannotated HR-TEM and FFT images corresponding to Figure 5d,e. These images of the GaN framework exclude grain color overlays and indexing. The FFT results confirm polycrystallinity, and IFFT overlays suggest no aggregation during TEM sampling.

**Figure S24.** Overview X-ray photoelectron spectroscopy (XPS) spectrum of bulk GaN and GaN framework.

**Figure S25.** X-ray photoelectron spectroscopy of early-stage fragmentation. Ga 3d XPS spectrum of the residual GaN framework after reacting in acetonitrile at 50 °C for 15 min.

**Figure S26.** Characterization of the GaN framework from the 1-octadecene control experiment. **a** Ga 3d X-ray photoelectron spectroscopy spectrum and **b** X-ray diffraction pattern of the residual GaN framework after heating and stirring in ODE.

**Figure S27.** High-resolution Ga 2p XPS spectra of bulk GaN and GaN framework. Binding energy analysis reveals a decrease in Ga–N bonding and an increase in Ga–O bonding. The enlarged Ga–O peak suggests that the GaN framework is more susceptible to surface oxidation compared to bulk GaN under identical drying conditions.

**Figure S28.** N 1s XPS spectrum of GaN framework. The N 1s signal overlaps with the Ga LMM Auger peak. No peak shift is observed in Ga LMM, whereas the N 1s peak intensity decreases slightly.

**Figure S29.** Identical-location TEM (ILTEM) tracking of structural evolution in GaN. **a**, TEM image of bulk GaN. **b**, FFT of (**a**). **c**, TEM image of the GaN framework. d, FFT of (**c**), showing different zone axes indicative of crystal transformation.

**Figure S30.** ILTEM evidence of fragmentation-induced zone axis rotation. TEM analysis confirms that two regions initially part of the same single crystal now show distinct zone axes in the GaN framework, supporting the occurrence of structural breakdown during fragmentation.

**Figure S31.** UV-vis absorption spectrum of GaN QDs from a 100-fold scaled-up synthesis.

**Table S1.** Crystalline domain sizes (Scherrer equation) of each facet for bulk GaN, GaN framework, and GaN QDs. Reduction in grain size for GaN framework

**Table S2.** Gaussian deconvolution of XPS spectra.
